# Effects of virtual reality-based exercise intervention in young people with attention-deficit/ hyperactivity disorder: a systematic review

**DOI:** 10.1186/s12984-025-01671-3

**Published:** 2025-06-23

**Authors:** Jia-Ling Sun, Xiao-Jun Chaw, Shane Fresnoza, Hsiao-I. Kuo

**Affiliations:** 1 School and Graduate Institute of Physical Therapy, National Taiwan University, No.17, Xu-Zhou Road, 10055 Taipei, Taiwan; 2 Department of Psychology, University of Graz, 8010 Graz, Austria

**Keywords:** Attention-deficit/hyperactivity disorder, Young people, Virtual reality, Exercise, Executive functions

## Abstract

**Background:**

Attention-deficit/hyperactivity disorder (ADHD) is a common neurodevelopmental disorder among the young population in the world. Young people with ADHD are often affected in their performance of attention, behavior, and executive functions (EFs), leading to a limited quality of life. Recently, Virtual reality (VR)-based exercise has been used as an intervention for young people with ADHD. Therefore, this review aims to evaluate the effectiveness of VR-based exercise in improving EFs and reducing ADHD symptoms in young people.

**Method:**

This review aims to systematically review the effects of VR-based exercise on the overall EFs and their subdomains, as well as ADHD symptoms in young people with ADHD. This review was registered in the International Prospective Register of Systematic Reviews (PROSPERO CRD42024604205) and was funded by the National Science and Technology Council, R.O.C., with the project number 112-2314-B002-119-MY3. Studies were identified in five databases (Cochrane Library, Web of Science, PubMed, SCOPUS, and Embase) from September 2010 through September 2024. Studies that applied VR-based exercise intervention on young participants with ADHD were included in this systematic review. A total of 6 studies met the inclusion criteria and were considered high quality according to standardized assessment lists.

**Results:**

Based on the 6 included studies and a total of 192 participants, the results showed that VR-based exercise with moderate to vigorous intensity provides positive effects on multiple subdomains of EFs (inhibitory control, attention, working memory, switching, and planning) and clinical symptoms in young people with ADHD. Furthermore, fully immersive and semi-immersive VR-based exercise interventions yielded similar results.

**Conclusion:**

VR-based exercise effectively improves EFs and is feasible for young people with ADHD, with benefits observed across ages and with sessions over 30 min. However, more evidence is essential for VR-based exercise intervention, which may compare the effects to other intervention types. Additionally, studies with rigorous experimental design are warranted.

## Background

Attention-deficit/hyperactivity disorder (ADHD) is a neurodevelopmental disorder characterized by behavioral symptoms including inattention, hyperactivity, and impulsivity. The global prevalence of ADHD among young people (children and adolescents) is between 5.6% and 7.6%, with an upward trend observed in recent years [1]. Aside from the core symptoms, such as inattention, hyperactivity, and impulsivity, executive function (EF) deficits are also easily seen in ADHD patients [2]. EF represents the higher-order neurocognitive processes, including working memory, inhibitory control, cognitive flexibility, organization, emotional control, task initiation, planning and prioritizing, self-monitoring, switching, and updating [3, 4]. During childhood and early adolescence, especially between the ages of 5 and 12, EFs would undergo rapid and foundational development, making this period a critical window for interventions targeting self-regulation and cognitive control in the ADHD population [5, 6]. Moreover, EF deficits might affect learning ability, social function, life achievement, and life quality in young people [7, 8]. Therefore, in the present study we focused the intervention for young people with ADHD.

Regarding ADHD intervention, many studies have reported medication as a first-line treatment [7, 9] because ADHD is mainly associated with neurotransmitter imbalance in the frontal and striatum [10, 11]. However, studies reported that around 10–30 % of young people have an inadequate response to pharmacotherapies, and some treated subjects suffer from side effects, including insomnia, loss of appetite, and headache [12, 13]. Additionally, it has been found that medication is less effective for EF for young patients [14]. Therefore, increasing efforts have been dedicated to probe the effects of non-pharmacological approaches on the young population. Of the different interventions, exercise has emerged as an effective and safe alternative way to improve clinical symptoms and EF in young people with ADHD [15]. Previous meta-analytic research has shown the beneficial effects of moderate to vigorous-intensity exercise on inhibitory control, cognitive flexibility performance, and behavior in young people [15]. Specific to the ADHD population, previous results showed significant improvement in inhibitory control, speed of processing, and attention after both acute and chronic aerobic exercise [16]. In addition to improving EFs, evidence has found that moderate to vigorous exercise significantly improved behavioral symptoms (e.g., impulsivity control and vigilance), academic outcomes, and quality of life in young people with ADHD [16–19].

Within the last decade, technological developments such as Internet-delivered strategy, assistive technology, and mobile applications, as well as exercise with virtual reality (VR), have raised great attention. VR is an advanced form of human-computer interactive technology that creates a sense of both presence and immersion. By enabling interactions between real and virtual environments, VR can enhance engagement through virtual agents [20, 21]. Furthermore, VR can be categorized into three types: fully immersive, non-immersive, and semi-immersive [22]. Fully immersive VR requires multiple devices, such as helmets, gloves, and sensory connectors, to create an encompassing experience; non-immersive VR, often viewed as basic VR, keeps a clearer separation between the virtual and real worlds; semi-immersive VR falls between these two, offering a balanced virtual experience. Increasingly, studies have shown that VR can be used in therapeutic settings, such as cognitive training, task-oriented training, and dual-task exercises, to treat neurodevelopmental disorders. VR-based interventions often require increased inhibition and task-switching components; therefore, they may boost an increased level of arousal, stimulate certain brain areas, and enhance EF performance in patients with ADHD [4]. Also, previous studies have proven that VR-based cognitive interventions are effective not only in improving working memory, attentional focus, and cognitive flexibility across various populations, but also in treating cognitive deficits in individuals with neurological diagnoses, although further research is needed to solidify these findings [23, 24]. Indeed, in line with this, studies have shown that VR-based interventions can effectively improve certain EF deficits in subjects with ADHD [20, 21]. Previous studies have also suggested that VR-based physical activity interventions may enhance engagement, multisensory stimulation, real-time feedback, and enjoyment among children with ADHD, thereby improving their participation in research activities [25, 26]. Nevertheless, systematic evidence to demonstrate the effects of VR-based exercise intervention on EFs in young people with ADHD is still rare. Thus, the aim of the study is to systematically review the effects of VR-based exercise on overall and subdomains of EFs and ADHD symptoms in young people with ADHD. Recent two systematic review articles have discussed some parts related to the topic [27, 28], however the review offers some different key points. First, the current work focused on VR-based interventions that include exercise, whereas the prior reviews included broader VR or simulator technologies. Second, the paper targeted specifically on young people, which allows for developmental relevance. Third, the study provided a more detailed analysis of executive function subdomains, rather than focusing only on attention or motor outcomes.

## Main text

### Methodology

#### Study design

This study was conducted according to the Preferred Reporting Items for Systematic Reviews and Meta-Analyses (PRISMA) guidelines [29] and was registered in the International Prospective Register of Systematic Reviews (PROSPERO CRD42024604205).

### Search strategy

A comprehensive search process was conducted on five different databases, including Cochrane Library, Web of Science, PubMed, SCOPUS, and Embase, with a publication date from September 2010 to September 2024. Table 1 shows the details of databases, search strategies, limits applied, and records identified. The main search categories were determined and applied in various ways: (1) ADHD, attention-deficit/hyperactivity disorder, attention deficit, or hyperactivity. (2) Children, adolescents, or young people. (3) Virtual reality-based exercise, VR-based exercise, exergam*, or serious gam*. (4) Cogniti*, executive function, EFs, behavior, behaviour, inhibition, inhibitory control, working memory, attention, cognitive flexibility, symptom, inattention, or hyperactivity. The publication range was restricted to studies published from 2010 onward, as most commercial and research-grade VR systems appropriate for pediatric exercise interventions, such as Nintendo Wii, HTC VIVE, became widely accessible only within the past decade.

**Table 1:** Search strategies

Database	Search strategies	Limits	Filter
Cochrane Library Web of Science PubMed SCOPUS Embase	((“ADHD” [Title] OR “attention deficit” [Title] OR “hyperactivity” [Title] OR "attention-deficit/hyperactivity disorder”[Title]) AND (“children” [Title] OR “adolescent” [Title] OR”young people” [Title]) AND (“virtual reality-based exercise” [Title] OR “VR-based exercise” [Title] OR “exergam*” [Title] OR “serious gam*” [Title]) AND (“cogniti*” [Title] OR “executive function” [Title] OR “EFs” [Title] OR “behavior” [Title] OR “behaviour” [Title] OR “inhibition” [Title] OR “inhibitory control” [Title] OR “working memory” [Title] OR “attention” [Title] OR “cognitive flexibility” [Title] OR “symptom” [Title] OR “inattention” [Title] OR “hyperactivity” [Title]))	Publication date from 2010/09/01 to 2024/09/01 - Humans - English language	13 items 12 items 35 items 53 items 28 items

### Eligibility criteria

The inclusion criteria for this systematic review are outlined in Table 2 using the framework of PICO. Studies that applied either acute or chronic VR-based exercise interventions to young participants with ADHD were included in this systematic review. The studies included were all available in full text and published in English. Studies without presenting results, such as clinical trial protocols and conference papers, were excluded. Further details concerning the eligibility criteria can be found in Table 2.

**Table 2:** Inclusion criteria of studies in the systematic review

PICO field	Criteria
Population	Participants were children and adolescents aged < 18 Participants who were diagnosed with attention-deficit/hyperactivity disorder by a board-certified psychiatrist according to the Diagnostic and Statistical Manual of Mental Disorders, Fifth Edition (DSM-V), the International Classification Diseases 10th Revision (ICD-10), or evidence-based questionnaires such as the Swanson, Nolan, and Pelham Questionnaire, Fourth edition (SNAP-IV), ADHD Rating Scale (ADHD-RS), Conners 3rd edition scale, and Conners’ Parent Rating Scale
Intervention	Either acute or chronic virtual reality-based exercise intervention. VR includes immersive and semi-immersive VR. Exercise indicates a physical activity that is planned, structured, repetitive, and purposive in the sense that improvement or maintenance of one or more components of physical fitness is an objective [30] Intervention such as VR-based cognitive training without combining exercise was excluded
Comparator	1. Studies using an inactive comparator (no treatment and waiting list), in which the participants received no treatment were included 2. Studies using an active comparator, in which the participants received a similar level of intervention with the participants in the intervention group, were included 3. Studies without a comparative group, in which the study design contains of only an intervention group, were also included
Outcome	1. Studies using standardized outcome measures to assess either the executive functions or ADHD symptoms of the participants. Examples of eligible outcome measures include Continuous Performance Test (CPT), Go/No-go test, Wisconsin Card Sorting Test (WCST), or SNAP-IV Questionnaire

### Data extraction

Three independent researchers extracted the information of title, aim, design, total sample size, population, type of intervention condition, outcome measures, confounders, main results, and conclusions from all studies. The results of extraction from each database were exported to EndNote Compress Library (version X9.2, Clarivate Analytic 2019). After the three researchers read all the titles and abstracts independently, a consensus meeting was conducted to unify the opinions. The latest review results were summarized initially, followed by a screening of studies that could be relevant to the chosen topics for retrieval.

### Risk of bias assessment

The quality appraisal for each study was independently conducted by two authors (SJL and CXJ) and subsequently reviewed by another author (KHI), using the Cochrane 'Risk of Bias 2' (RoB 2) tool [31]. Based on the RoB 2 tool criteria, studies were categorized into three risk levels: low risk of bias, high risk of bias, and some concerns. The assessments covered five domains: bias arising from the randomization process, bias due to deviations from the intended interventions (effect of adhering to intervention), bias due to missing outcome data, bias in measurement of the outcome, bias in selection of the reported result, and an overall bias.

## Results

### Study selection

We identified 141 records through databases with no additional records from registers or other organizations. After excluding 58 duplicates, we screened 83 records by title and abstract and eliminated 61 records. Afterward, we attempted to retrieve the remaining 22 studies, excluding 3 more in the process. Finally, we conducted a full assessment of the 19 remaining records, excluding 13 studies due to differing outcomes or interventions, resulting in 6 studies for inclusion.

### General findings

**Fig. 1:**
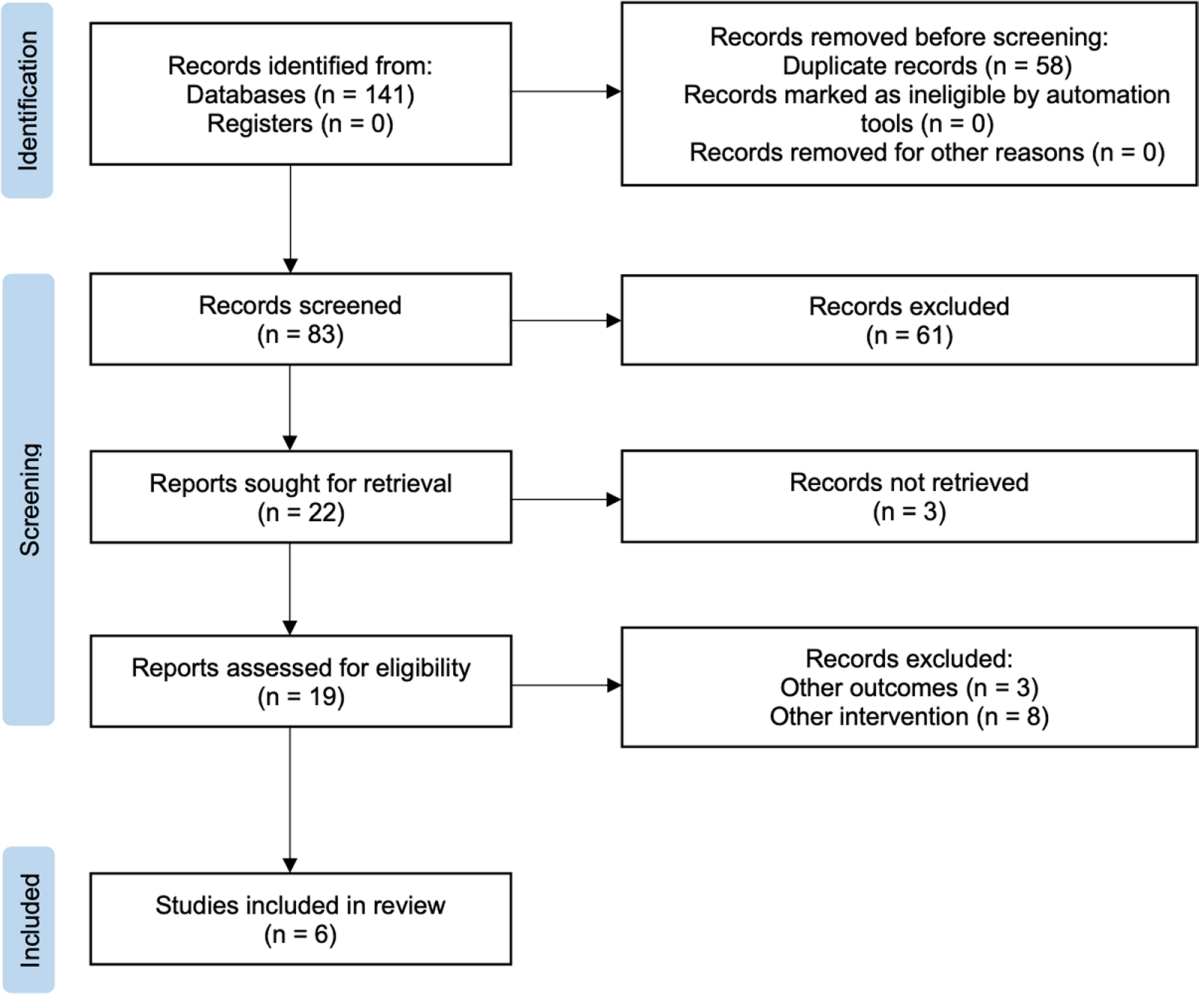
Flow of articles through the search process

Figure 1 illustrates the flow of search results throughout the systematic review process, ultimately resulting in the inclusion of 6 articles. Upon analysis, it was found that one study (17%) investigated acute exercise interventions [32], while the remaining five (83%) focused on chronic exercise interventions [4, 26, 33–35]. All studies employed group-randomized controlled trials, except for Ou et al. [35], which was a case series involving 3 participants. The review analyzed data from 192 participants, with sample sizes ranging from 3 [35] to 51 [4]. Participants had similar medication statuses: in five studies, some of the participants were on medication [4, 26, 32, 33, 35]. While in one study, none of the participants were on medication [34]. A detailed overview of all the studies is provided in Table 3.

**Table 3:** Characteristics of the analyzed studies (n = 6)

Author	Study design	Participants	On medication	Measures	Intervention	Control/comparison	Measuring timepoints	Outcome
Benzing et al. (2018) [32]	RCT	46 children with ADHD (8–12 years, 38 boys and 8 girls): 24 in intervention group, 22 in control group	Partially (36 out of 46 participants)	Modified Flanker test (measuring inhibition and switching) and modified Color Span Backward test (measuring visual working memory)	Semi-immersive exergaming with “Xbox Kinect” in moderate to vigorous intensity; one-bout; 15 min	Inactive (watching a documentary report about mountain running)	Pre- and immediately post-intervention	*Flanker test* Significantly decreased reaction time in inhibition and switching task; no significant difference in accuracy *Color Span Backward test* No significant difference
Benzing et al. (2019) [4]	RCT	51 children with ADHD (8–12 years, 42 boys and 9 girls): 28 in intervention group, 23 in control group	Partially (37 out of 51 participants)	Modified Simon task (measuring inhibition), modified Flanker test (measuring switching), modified Color Span Backward test (measuring updating), ADHD symptoms, and motor ability	Semi-immersive exergaming with “Xbox Kinect” in moderate to vigorous intensity; 8 weeks; 3x/week; 30 min/session	Inactive (in a waiting-list)	Pre- and post-intervention (after 8 weeks)	*Simon task* Significantly decreased reaction time; no significant difference in accuracy *Flanker test* Significantly decreased reaction time; no significant difference in accuracy *Color Span Backward test* No significant difference *ADHD symptoms* Significantly decreased global index score; no significant difference in DSM‐IV symptom scales *Motor ability* significantly better total performance
Chang et al. (2022) [33]	RCT	48 children with ADHD (7–12 years, 39 boys and 9 girls): 16 in intervention group, 16 in active control group, and 16 in inactive control group	Partially (12 out of 48 participants)	Stroop test (measuring sustained visual attention), Wisconsin Card Sorting Test (WCST) (measuring planning and inhibition), and Graphomotor Function Tests (measuring handwriting performance)	Semi-immersive simulated table tennis with “Nintendo Wii Sport”; 12 weeks; 3x/week; 60 min/session	Actual table tennis; 12 weeks; 3x/week; 60 min/sessions Inactive (not receiving training)	Pre- and post-intervention (after 12 weeks)	*Stroop test* Both simulated and actual table tennis groups showed a significant improvement in Stroop Color-Word Test, especially the simulated table tennis group; no significant difference in Stroop Color Test *WCST* Actual table tennis group showed a significant improvement in non-perseverative and total errors *Graphomotor Function Tests * Both simulated and actual table tennis groups showed a significant increase in short paragraph copy speed and stroke velocity, and a significant decrease in reaction time and time to automation
Ji et al. (2023) [26]	Non-RCT	30 children with ADHD (8–12 years, 26 boys and 4 girls): 21 in intervention group, 21 in control group	Partially	Go/No-go test (measuring inhibitory control), Frankfurter Aufmerksamkeits-Inventar (FAIR) (measuring attention), and frontal lobe EEG during Go/No-go test	Semi-immersive exergaming with “ExerHeart” in moderate to vigorous intensity; 4 weeks; 3x/week; 50 min/session	Stationary bicycle exercise in moderate to vigorous intensity; 4 weeks; 3x/week; 50 min/session	Pre- and post-intervention (after 4 weeks)	*Go/No-go test* Both groups showed a significant decrease in reaction time *FAIR test* Both groups showed a significant improvement in selective attention, self-control, and persistent attention *Frontal lobe EEG * Significantly increased Go N2 amplitude
Shiratzky et al. (2019) [34]	Noon-RCT	14 children with ADHD (8–12 years, 11 boys and 3 girls)	No	Conners’ Parent Rating Scale-Revised (measuring behavior), the NeuroTrax™ computerized neuropsychological battery test (measuring attention and memory), and dual task	Semi-immersive VR-based treadmill training in a graduate progressed intensity; 6 weeks; 3x/week; 30–60 min/session	NA	Pre-, post-intervention, and 6-week follow-up	*Conners’ Parent Rating Scale-Revised* Significantly improved social problems and psychosomatic behavior *NeuroTrax™* Significant improvement in memory, orienting attention, and sustained attention; no significant difference in sustained attention Dual task Significantly improved step and stride regularity
Ou et al. (2020) [35]	Case series	3 children with ADHD (8–12 years, 1 boy and 2 girls)	Partially (2 out of 3 participants)	Test of Nonverbal Intelligence (TONI-4), Attention Test for Elementary School Children (ATESC), WCST (measuring cognitive flexibility), and parent form SNAP-IV	Immersive exergaming with “HTC VIVE” in a graduate progressed intensity; 12 weeks; 3x/week; 40 min/session	NA	Pre- and post-intervention (after 12 weeks)	*TONI-4* Improved score *ATESC* Improved score *WCST* Improved score in one participant, with diminished performance in the other two *SNAP-IV* Decreased scores in inattention, hyperactivity/ impulsivity, and oppositional defiant disorder

*RCT* Randomized controlled trial, *ADHD* Attention-deficit/hyperactivity disorder, *DSM‐IV* Diagnostic and Statistical Manual of Mental Disorder, Fourth edition, *EEG* electroencephalogram, *NeuroTrax™ *NeuroTrax Corp., Medina, NY. *SNAP-IV *Swanson, Nolan, and Pelham Questionnaire, Fourth edition

### Measurements of executive functions

All six studies analyzed the effects of VR-based exercise training on executive functions. Four of the studies examined inhibitory control using various tasks, including the Modified Flanker test, Modified Simon task, Wisconsin Card Sorting Test (WCST), and the Go/No-go test [4, 26, 32, 33]. Among the four studies, Benzing et al. [32], Benzing et al. [4], and Ji et al. [26] found a significant reduction in reaction time during the tasks, with no significant differences in accuracy. On the other hand, Chang et al. [33] reported that participants in the actual table tennis group showed a greater improvement in non-perseverative errors and total errors on the WCST compared to those in the simulated table tennis group.

Four studies assessed attention performance using the Stroop test, the Frankfurter Aufmerksamkeits-Inventar (FAIR) test, and the Attention Test for Elementary School Children (ATESC) [26, 33–35]. Both Chang et al. [33] and Ji et al. [26] reported significant improvements in attention for participants in both the VR-based exercise group and the exercise group. Specifically, Chang et al. [33] observed more significant improvement in the simulated table tennis group, while Ji et al. [26] found significant enhancements in selective attention, self-control, and sustained attention in both groups. Additionally, Ou et al. [35] reported improved scores in five attention dimensions—focused, sustained, selective, alternating, and divided—following the VR-based exercise intervention. Shiratzky et al. [34] assessed multiple dimensions of attention, including sustained, orienting, and selective attention. After the VR-based treadmill training, participants showed improvements in orienting and selective attention, while no significant differences were observed in sustained attention.

Working memory performance was evaluated in two studies using the Color Span Backward test and the NeuroTrax™ computerized neuropsychological battery [32, 34]. Benzing et al. [32] found no significant differences in working memory after the VR-based exercise intervention, whereas Shiratzky et al. [34] reported a significant improvement following VR-based treadmill training. Additionally, two studies assessed switching performance using the Modified Flanker test [4, 32]. Both studies reported a significant decrease in reaction time with no significant differences in accuracy. Cognitive flexibility was examined by Ou et al. [35] using the WCST, with results showing an improved score in one participant and diminished performance in the other two. Benzing et al. [4] assessed the updated performance with the modified Color Span Backward test, which showed no significant difference following the VR-based exercise intervention. Lastly, Chang et al. [33] evaluated planning performance through the WCST, finding a significant improvement in non-perseverative and total errors only in the actual table tennis group.

In conclusion, all six studies reported improvements in EFs in different domains. Table 4 shows that there are three out of four studies reported improvements in inhibitory control; four out of four studies reported improvements in several domains of attention; one out of two studies reported improvements in working memory; and one out of one study reported improvement in planning.

**Table 4:** EF differences after VR-based exercise intervention

	Inhibitory control	attention	Working memory	switching	Cognitive flexibility	updated	planning
Benzing et al. (2018)	+		**−**	+			
Benzing et al. (2019)	+			+		**−**	
Chang et al. (2022)	**−**	+					+
Ji et al. (2023)	+	+					
Shiratzky et al. (2019)		**+ **	** + **				
Ou et al. (2020)		+			**−**		

+: EFs improved after VR-based exercise intervention; −: EFs remained the same or declined after VR-based exercise intervention

### Measurements of ADHD symptoms

Three studies evaluated ADHD symptoms following the VR-based exercise intervention [4, 34, 35], all of which reported an improvement in ADHD symptoms after the intervention. Specifically, Benzing et al. [4] analyzed ADHD symptoms through the Conners 3rd edition scale (Conners-3) and the Diagnostic and Statistical Manual of Mental Disorders (DSM‐IV‐TR) Symptom Scales. The results showed a significant decrease in the global index score on the Conners-3 scales, with no significant differences in the DSM‐IV‐TR subscales, including inattentiveness, hyperactivity, and combined. Shiratzky et al. [34] observed significant improvements in social problems and psychosomatic behavior using the revised version of the Conners’ Parent Rating Scale-Revised (CPRS-R). Ou et al. [35] reported decreased scores in the SNAP-IV questionnaire for inattention, hyperactivity/impulsivity, and oppositional defiant disorder subscales.

### Comparison between VR-based exercise and traditional exercise intervention

Among the six included studies, two studies [26, 33] included active control groups using traditional exercise, allowing for a direct comparison with VR-based interventions. In Chang et al. [33], both actual table tennis (traditional exercise) and simulated table tennis (VR-based exercise) led to significant improvements in EFs and handwriting performance, though only the actual table tennis group demonstrated a significant improvement on the WCST. In Ji et al. [26], both the exergaming group and the bicycle exercise group showed improvements in attention-related behavioral outcomes,however, only the exergaming group exhibited a significant increase in N2 amplitude on the Go/No-go task, indicating enhanced neural correlates of attention. These findings suggest that while both traditional and VR-based exercises can benefit EFs and attention, VR-based interventions may offer additional neurocognitive engagement in specific domains.

Regarding adherence, Ji et al. [26] also noted differences in engagement, with all dropouts in the bicycle exercise group attributed to loss of interest, while dropouts in the exergaming group were due to scheduling conflicts, suggesting that VR-based exercise may enhance motivation and adherence in children with ADHD.

### Other findings

Aside from executive functions and ADHD symptoms, studies also found other benefits of VR-based exercise intervention. Benzing et al. [4], Chang et al. [33], Shiratzky et al. [34], and Ou et al. [35] found a significantly improved motor function in young ADHD participants after the VR-based exercise intervention. First, Benzing et al. [4] evaluated the motor abilities of the participants through the German Motor Test. Results showed that participants in the VR-based exercise group showed significantly better motor performance than the control group, especially in two items: jumping sideways and push-ups. Second, Chang et al. [33] found that both simulated and actual table tennis training led to improvements in the handwriting performance of the participants. To successfully hit the ball, participants must concentrate on it, enhancing their eye-tracking and eye-hand coordination abilities. This process trains executive functions, such as attention, while also improving eye-hand coordination, which can lead to better handwriting performance and learning fluency. Third, Shiratzky et al. [34] evaluated the step and stride regularity of participants and observed significant improvement immediately after VR-based treadmill training. Although this improvement was not maintained at the 6-week follow-up, there was still a trend of improvement at that time point. Following the VR-based treadmill training, the walking patterns of the participants became more consistent, suggesting more mature motor control. Lastly, Ou et al. [35] found that after VR-based exercise training, participants showed improvements in eye-hand and hand-foot coordination. This may be due to the engagement of both upper and lower extremities during the VR-based training sessions.

Shiratzky et al. [34] evaluated the behavior of the participants through the long form of the Conners’ Parent Rating Scale-Revised (CPRS-R). Results showed that the social problems, including difficulty in making new friends or maintaining relationships with friends, were reduced after applying VR-based treadmill training in young people with ADHD. While the improvement was not fully sustained at the 6-week follow-up, a positive trend was still observed at the time point. Additionally, the psychosomatic behavior, such as frequent complaints about pain and fatigue, of the ADHD participants was also improved after the VR-based treadmill training and even persisted in the 6-week follow-up time point. These findings indicated that VR-based exercise training might have a positive impact on several features of behavior and is a potential tool for the treatment of ADHD.

### Risk of bias assessment

In terms of quality assessment, all 6 studies were assessed to be of high quality. All the studies except Shiratzky et al. [34] and Ou et al. [35] were judged to be at low risk of bias in the randomization process. Shiratzky et al. [34] and Ou et al. [35] have only one experimental design, lacking a control group,therefore, no randomization process was conducted. Except for Shiratzky et al. [34] and Ou et al. [35], all studies were considered to have a low risk of bias due to deviations from the intended intervention (effect of adhering to intervention). Although there were some dropouts in the studies, the dropout rates across all groups were considered similar in every study, and there were no statements indicating the assessments addressed imbalances in non-protocol interventions between the intervention groups. All the studies except Ji et al. [26] were considered to have a low risk of bias related to missing data, as outcome data was available for all or nearly all participants after the whole intervention process. Similarly, all studies were rated as having a low risk of bias in outcome measurement. However, due to the lack of pre-registration and limited available information, all studies were classified as having some concerns about the risk of bias for selective reporting. Overall, all the studies were determined to have an unclear risk of bias. Table 5 provides a summary of the risk of bias assessments for each study, categorized into three levels: low, high, and some concerns.

**Table 5:** Risk of bias assessment for the included studies

Author	Randomization process	Deviations from the intended interventions	Missing outcome data	Measurement of the outcome	Selection of the reported result	Overall risk of bias
Benzing et al. (2018)	Low	Low	Low	Low	Some concerns	Some concerns
Benzing et al. (2019)	Low	Low	Low	Low	Some concerns	Some concerns
Chang et al. (2022)	Low	Low	Low	Low	Some concerns	Some concerns
Ji et al. (2023)	Low	Low	Some concerns	Low	Some concerns	Some concerns
Shiratzky et al. (2019)	Some concerns	Some concerns	Low	Low	Some concerns	Some concerns
Ou et al. (2020)	Some concerns	Some concerns	Low	Low	Some concerns	Some concerns

## Discussion

### Main findings

This systematic review has investigated the effects of VR-based exercise intervention on EF and ADHD symptoms in young people with ADHD. The main results showed that VR-based exercise improves EF with inhibitory control, attention, working memory, switching, and planning, whereas it showed no differences in cognitive flexibility and updating. Additionally, ADHD symptoms, motor ability, and behavior were improved after applying VR-based exercise intervention.

Among all the studies that have applied VR-based exercise interventions, only one study was conducted in a single session (15 min/ session) [32],the other five studies were all conducted in long-term sessions ranging from 4 to 12 weeks (moderate to vigorous intensity, 30–60 min/session, 3 sessions/week). The results of the EFs were similar between acute and chronic conditions, except for working memory. Here, Benzing et al. [32] assessed working memory by applying the Color Span Backward test and found no significant differences between intervention and control groups. Regarding the lack of effects on working memory, the study suggested that the 15-min duration of the one-bout VR-based exercise might be too short, which provides an insufficient dosage to enhance working memory performance.

Four studies have evaluated participants' performance in inhibitory control [4, 26, 32, 33], all reporting improvements after the application of VR-based exercise interventions. In previous research, inhibitory control has been assessed using various tasks, including the Stroop test [36, 37], Go/No-Go test [38, 39], Eriksen Flanker test [18], and the Continuous Performance Test [40]. Most of these studies observed a moderate effect size following exercise interventions, consistent with the findings of the four studies included in this systematic review. Furthermore, a prior meta-analysis also highlighted that the most substantial effects of exercise appear to be on inhibition [41]. This may be explained by the activation of prefrontal and fronto-striatal circuits during exercise, which are particularly involved in self-regulatory processes. Supporting this, Ji et al. [26] reported an increase in N2 amplitude during a Go/No-go task following exergaming, suggesting enhanced neural responsiveness in regions underlying inhibitory control.

Attention has been evaluated in four studies in the present review [26, 33–35]. All reported improvements following VR-based exercise interventions in specific aspects. Chang et al. [33], Ji et al. [26], and Ou et al. [35] observed significant improvements in sustained attention. Shiratzky et al. [34] and Ou et al. [35] reported significant improvements in participants’ selective attention after the intervention. Previous research suggests that exercise can enhance cerebral blood flow and stimulate the secretion of catecholamines, which could improve information processing speed and attention performance in children with ADHD [38, 42, 43]. Additionally, the integration of VR applications may amplify these effects, further enhancing various aspects of attention in children with ADHD.

Three studies included in this systematic review evaluated participants’ ADHD symptoms [4, 34, 35]. All reported significant improvements in specific subscales. Benzing et al. [4] observed a significant reduction in the global index score of the Conners-3, indicating an improvement in symptoms from 'clinically elevated' to 'borderline'. The finding aligns with previous studies, which found moderate-to-vigorous physical exercise programs significantly improved social problems and psychosomatic behavior subscales in young people with ADHD. Furthermore, the effect persists to a six-week follow-up. Similarly, Ou et al. [35] evaluated participants’ ADHD symptoms using the SNAP-IV rating scale and found significant improvements across all three criteria: inattention, hyperactivity/impulsivity, and oppositional defiant disorder, indicating an overall improvement in their ADHD symptoms after exercise intervention.

This systematic review included studies utilizing both fully immersive VR [35] and semi-immersive VR [4, 26, 32–34] for VR-based exercise interventions. The review found that the effects of VR-based exercise on EFs and symptoms are independent of the types of VR. However, this may be due to the limited number of studies and small sample sizes. Further research with larger samples is needed to determine whether the level of immersion influences cognitive or behavioral outcomes in children with ADHD. One issue that should be taken into consideration is the potential adverse effects of VR-based exercise intervention. However, a recent systematic review examining the effectiveness of immersive VR for addressing cognitive deficits in children with ADHD considered potential adverse events associated with immersive VR [20]. Among the two studies that evaluated adverse events during immersive VR interventions, neither reported moderate or severe simulator sickness symptoms (e.g., general discomfort, fatigue, headaches, eye strain, stomach awareness, nausea, dizziness, vertigo, sweating, blurred vision, and other related issues) [44, 45].

### Clinical implications

Key findings from the current review suggested that VR-based exercise intervention can be used as an alternative tool to improve global EF, including inhibitory control, attention, working memory, switching, and planning in young people with ADHD. However, more studies are warranted since the results are still controversial. The most pivotal issue is whether improvements in EF can translate to real life. Results seem to suggest that the effects are clinically meaningful. Outcomes such as improved handwriting [33], faster reaction times [26], and improved parent/teacher-rated behavior [4] may reflect changes with meaningful educational or behavioral benefits. Moreover, exercise interventions that incorporate cognitive demands, such as those using VR, have been shown to produce more significant and longer-lasting improvements in several subdomains of EFs compared to traditional exercise [46]. We have recommended that future studies incorporate real-world outcome measures to enhance interpretability and impact. Moreover, improvements in EFs for young people with ADHD were observed across all ages, and the intervention duration over 30 min was also proven to be effective. Most importantly, the current review supports the feasibility of VR-based exercise intervention.

### Limitations

In spite of the significant findings of the study, there are some limitations of the current study. First, the review included a limited number of eligible studies, which led to limited comparable data on different measures of a construct. This may cause a large heterogeneity and publication bias. As less than 10 studies were included in the review, the publication bias might not be properly assessed for some outcome measures. Furthermore, considering the limited number of studies included in this systematic review and because only a few outcome measures were consistently reported across the included studies, we did not conduct a meta-analysis to synthesize the results of the studies. The finding of this study is considered preliminary evidence for the effects of VR-based exercise in young people with ADHD. Second, the cautions interpreting results of the study are due to the limitations reported by the recruited studies regarding study design, insufficient background information from the participants (medication use, ADHD subtypes, symptom severity, family factors), lacking active control group, and intervention intensity. Third, the studies selected for review were limited because only those with specific terms mentioned in the title/abstract were screened, and those non-English published studies were not included. Fourth, only one study [34] assessed the long-term performance of the participants with a 6-week follow-up. Without sufficient long-term assessments, it remains unclear whether the cognitive and behavioral improvements observed from VR-based exercise interventions are sustained over time. Finally, the heterogeneity of outcome measurements used across the included studies reflects the broad and multidimensional structure of EF. However, this variation makes it difficult to compare results among the studies. Future research would benefit from using standardized cognitive assessments and including both behavioral and neurophysiological outcomes to improve comparability and synthesis.

Aside from the above limitations, none of the included studies conducted formal statistical analyses to identify mediators or moderators, several variables varied across studies and may have influenced intervention outcomes. First, the level of VR immersion differed. Ou et al. [35] employed a fully immersive VR intervention using a head-mounted display, whereas the remaining studies used semi-immersive VR. Second, intervention duration and intensity ranged from a single bout 15-min session [32] to long-term protocols lasting up to 12 weeks with three sessions per week [33], potentially moderating the magnitude of cognitive and behavioral changes. Third, all studies focused on children aged approximately 7 to 12 years, but participant age was not examined as a subgroup variable, despite possible developmental differences in responsiveness to training. Fourth, medication status was inconsistently reported and not systematically analyzed. For example, except Shiratzky et al. [34], all the other studies included some participants who continued ADHD medication use during the study period. Lastly, the type of control group also varied. Chang et al. [33] used both active and inactive control groups, whereas other studies relied solely on inactive or no control conditions. These unexamined factors may have influenced study results and should be considered in future trials.

## Conclusion

In summary, this review has demonstrated that VR-based exercise appears applicable in improving EF and ADHD symptoms, though results are mixed and limited by study heterogeneity. While outcomes were comparable between VR-based and traditional exercise in some studies, VR-based approaches may offer additional benefits in terms of neural activation and participant engagement, making them a promising and motivating intervention option. The findings provide preliminary evidence and highlight the need for more high-quality RCT studies with clear reporting of methodology and a more detailed analysis of potential mediators or moderators, which will allow further reviews to draw clear and confident conclusions regarding the effectiveness of VR-based exercise intervention in clinical application in young people with ADHD.

## Data Availability

No datasets were generated or analysed during the current study.

## Abbreviations

ADHD: Attention-deficit/hyperactivity disorder
ATESC: Attention Test for Elementary School Children
CPT: Continuous Performance Test
Conners-3: Conners third edition scale
CPRS-R: Conners’ Parent Rating Scale-Revised
DSM-V: Diagnostic and Statistical Manual of Mental Disorders, Fifth Edition
DSM-IV: Diagnostic and Statistical Manual of Mental Disorders, Fourth Edition
EF: Executive function
EEG: Electroencephalogram
FAIR: Frankfurter Aufmerksamkeits-Inventar test
HQ: High quality
ICD-10: International Classification Diseases 10th Revision
LQ: Low quality
MQ: Medium quality
PRISMA: Preferred Reporting Items for Systematic Reviews and Meta-Analyses
RCT: Randomized controlled trial
SNAP-IV: Swanson, Nolan, and Pelham, Version IV
TONI-4: Test of Nonverbal Intelligence
VR: Virtual reality
WCST: Wisconsin Card Sorting Test
